# Metapopulation Dynamics of the Mistletoe and Its Host in Savanna Areas with Different Fire Occurrence

**DOI:** 10.1371/journal.pone.0065836

**Published:** 2013-06-11

**Authors:** Grazielle Sales Teodoro, Eduardo van den Berg, Rafael Arruda

**Affiliations:** 1 Departamento de Botânica, Instituto de Biologia, Universidade Estadual de Campinas, Campinas, Brasil; 2 Setor de Botânica Sistemática, Departamento de Biologia, Universidade Federal de Lavras, Lavras, Brasil; 3 Instituto de Ciências Naturais, Humanas e Sociais, Universidade Federal de Mato Grosso, Sinop, Brasil; DOE Pacific Northwest National Laboratory, United States of America

## Abstract

Mistletoes are aerial hemiparasitic plants which occupy patches of favorable habitat (host trees) surrounded by unfavorable habitat and may be possibly modeled as a metapopulation. A metapopulation is defined as a subdivided population that persists due to the balance between colonization and extinction in discrete habitat patches. Our aim was to evaluate the dynamics of the mistletoe *Psittacanthus robustus* and its host *Vochysia thyrsoidea* in three Brazilian savanna areas using a metapopulation approach. We also evaluated how the differences in terms of fire occurrence affected the dynamic of those populations (two areas burned during the study and one was fire protected). We monitored the populations at six-month intervals. *P. robustus* population structure and dynamics met the expected criteria for a metapopulation: i) the suitable habitats for the mistletoe occur in discrete patches; (ii) local populations went extinct during the study and (iii) colonization of previously non-occupied patches occurred. The ratio of occupied patches decreased in all areas with time. Local mistletoe populations went extinct due to two different causes: patch extinction in area with no fire and fire killing in the burned areas. In a burned area, the largest decrease of occupied patch ratios occurred due to a fire event that killed the parasites without, however, killing the host trees. The greatest mortality of *V. thyrsoidea* occurred in the area without fire. In this area, all the dead trees supported mistletoe individuals and no mortality was observed for parasite-free trees. Because *P. robustus* is a fire sensitive species and *V. thyrsoidea* is fire tolerant, *P. robustus* seems to increase host mortality, but its effect is lessened by periodic burning that reduces the parasite loads.

## Introduction

Mistletoes are perennial and aerial hemiparasitic plants. The majority of mistletoe in Brazil belongs to the family Loranthaceae [1]. These species attach to branches and trunks of host plants by a haustorial connection [2] and take water, photosynthates and mineral nutrients from their hosts [3], [4]. They are categorized as hemiparasites because they can carry on photosynthesis [4]. These plants are dispersed primarily by frugivorous birds; dispersal is a critical event for the parasites, since their seeds need to be deposited on the branches and trunks of host plants for successful colonization [5], [6]. Moreover, the distribution of these plants depends on their own physiological tolerance to abiotic factors, since some hemiparasite species are susceptible to frost and fire [7], [8].

Fire is considered the main natural control agent of mistletoe species in many ecosystems [9]. Historical fire regimes has been an important factor to determinate the distribution and abundance of North American mistletoe, while effective suppression of wildfires has contributed to an increase the abundance of mistletoe population in these forests in North America [9]. In Australia, changes in fire regimes are affecting spatial patterns of mistletoe diversity and abundance [10]. In Brazilian savannas, Fadini & Lima (2102) showed that the fire effects on the population vary with the mistletoe species, even for sympatric ones [8].

Mistletoes occupy a habitat with a distinct spatial structure composed by a collection of patches susceptible to colonization (host trees) in a matrix (area among the trees) or trees not susceptible to colonization (non-host trees) [11]. Dispersion occurs within and among habitat patches [11], [12] and due to this structure can be viewed as metapopulation [11]. A metapopulation is defined as a set of populations occupying discrete habitats patches, where each patch has asynchronous local dynamics [13], [14]. Persistence of the metapopulation dynamics is a result of the balance between local extinction of populations in patches and colonization processes of unoccupied habitats patches [15], [16], [17]. Trees are habitat patches for various organism groups such as insects, lichens, fungi, mosses, other epiphytes and mistletoe [18], [19], [20]. Trees as habitat patches are dynamic, because they emerge, grow and die [18], [21], and the ability of species to colonize and remain in these habitat patches is significantly dependent on the tree dynamics [21], [22], [23].

Freckleton and Watkinson [17] did not consider the system formed by mistletoes and their host trees as metapopulations because, in their understanding, the metapopulation concept only applies to regional scales. That position was opposed by Ehrlén and Eriksson [24] as being too restrictive. As those last authors argue, if the processes that govern a population fit to the metapopulation theory, then, the metapopulation framework should be used. Mistletoe populations seem to fulfill the four conditions stated by Hanski [14] in the incident function model and supported by Freckleton and Watkinson [17], [25] for existence of a metapopulation: (i) their suitable habitat “occur in discreet patches (host trees) that may be occupied by local reproducing populations”, (ii) their subpopulations have a measurable risk of extinction, (iii) their habitat patches (host trees) are not too isolated to prevent recolonization after local extinction, and (iv) their local populations have completely asynchronous dynamics.

One of most constraining issues for a correct metapopulation approach is the difficulty to define suitable habitat patches [17]. This issue is easily solved for mistletoes in general and negligible for those specialized in some host species. Therefore, considering everything, mistletoes’ populations seem to be good model systems for investigating the mechanisms and processes that create patterns in the plant metapopulation structure [26], [27]. Thus, agreeing with Overton [9], we opted to approach our studied mistletoe populations using the metapopulation framework. A similar approach was used in several studies with species of epiphytic bryophytes in boreal forests modeled the metapopulation dynamics in habitat patches [18], [22], [23], [28], [29], [30], [31].

We evaluated the dynamics of the mistletoe *Psittacanthus robustus* Mart. and its host *Vochysia thyrsoidea* Pohl. in three savanna areas with different fire occurrences. We sought to answer the following questions: i) do the dynamics of the *P. robustus* local populations on *V. thyrsoidea* trees fit to the expected metapopulation model? ii) does the mistletoe occupancy affect the dynamics of the habitat patches (trees)? iii) are patch extinction patterns similar among the non-burned area and the two burned areas? We expected that: i) the mistletoe local populations fit in a metapopulation model; ii) the mistletoe occupancy affects the dynamics of the host specie (habitat patch) due the parasitism effect on the host survivorship; iii) since some mistletoe species are sensible to fire, we expect greater changes in mistletoe populations in sites burned than in the non-burned ones.

## Materials and Methods

### Study Area

We conducted this study in three Brazilian savanna areas; two of them are recognized as rock outcrop savanna and one as *sensu stricto* savanna. The first area is located in the ‘Parque Ecológico Quedas do Rio Bonito’ (PEQRB), in the municipality of Lavras, Minas Gerais state (Brazil). The local climate, according to Köppen classification, is transitional between Cwb and Cwa, with average annual precipitation of 1530 mm and average annual temperature of 19.4°C. Altitudes range from 950 to 1200 m [32]. This area is an outcrop savanna [33], with its center located at coordinates 21°19′45,31″S and 44°58′22,69″W, and has been protected from fire for at least 15 years.

The second and third areas are in the municipality of Carrancas, Minas Gerais state, Brazil. Fires frequently occur in these sites. The local climate, according to Köppen classification, is Cwa, with average annual temperature of 14.8°C and average annual precipitation of 1490 mm. The study area ‘Carrancas-Zilda’ (CZ) is a physiognomy of *sensu stricto* savanna [33] on cambisol (the coordinates are 21°28′16″S and 44°37′21″W). This site was burned at 2008, between the first and second survey. The area ‘Carrancas-Esmeralda’ (CE) is an outcrop savanna [33], which the coordinates are 21°27′59″S e 44°42′10″W. This area was burned a year before the beginning of the study (2007).

### Studied Species

*Psittacanthus robustus* Mart. (Loranthaceae) is a neotropical hemiparasite that mainly colonizes Vochysiaceae species in savanna communities of Brazil [5], [6], [34]. Like other hemiparasitic species, its seeds have a mucilaginous and adherent substance in its apical region, which facilitates adhesion to the host [5]. *P. robustus* bloomed from November to March (rainy season) [35], but in the studied sites the individuals blossomed over the year, with a flowering peak in rainy season (personal communication GS Teodoro).

*Vochysia thyrsoidea* Pohl (Vochysiaceae) is the main host of *P. robustus* in the study areas [32]. This is characteristic tree of the cerrado that accumulates aluminum, giving it a competitive advantage in the acid soils of the cerrado (rich in aluminum) [33]. The species average height in the study areas was 5.50 meters. The pattern of spatial distribution for *V. thyrsoidea* was random in the PEQRB area and clumped in the areas CE and CZ (data not shown).

### Data Collection

We sampled 2.8 hectares in each studied area. In those areas, we mapped all individuals of *V. thyrosidea* (habitat patches) with height equal or higher than two meters and recorded all individuals of *P. robustus* on those trees. Individuals of *P. robusuts* are easily detected on their host and we marked their position in each tree and followed their dynamics during the study.

We monitored the populations of *P. robustus* and *V. thyrsoidea* at six month intervals. In the PEQRB area, we conducted six surveys (June 2007 to September 2009) and, for the areas CE and CZ, we had three surveys in each (February 2008 to August 2009). We followed the colonization, re-colonization and extinction of mistletoe in each habitat patch (individuals of *V. thyrsoidea*). We also evaluated the losses, gains and persistence of occupied and no-occupied patches (trees of *V. thyrsoidea*).

### Analyses

We used the four criteria state by Hanski [14] to test if the *P. robustus* dynamics fit to the metapopulation model and to discuss the differences found for the studied populations due to contrasting fire occurrence among sites. We calculated the metapopulation parameters for patch dynamics based on Overton [11]. The habitat patches had their own intrinsic turnover rate and, therefore, we separated the extinction parameter (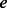) into two components:
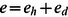
(1)


Where: 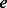
represents the extinction parameter; 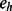 is the number of local populations on patches lost due to demographic or exogenous causes; 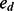 represents the loss of local population due to patch extinction. This distinction between the components of extinction is relevant in our study, because is related to the contrasting fire events among areas.

We also calculated the balance – rate of equilibrium proportion occupied (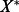) – between new patches colonized by mistletoe and patches where the mistletoe populations went extinct:
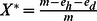
(2)


Where: 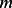 is the number of new patches colonized. 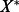 will assume positive values for 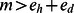 *;* and negative values for 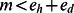 [11].

We also analyzed the host and mistletoe population dynamics based on Sheil et al. [36], [37] that consider changes in population size per time interval in a size-constant proportion. For the mortality rates, the initial population size was the reference and, for recruitment, the reference was the final size. We calculated the rates of mortality (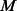), recruitment (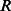) and net change rate (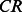) for populations of *V. thyrsoidea* and *P. robustus*:
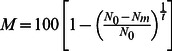
(3)

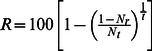
(4)

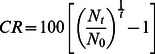
(5)


Where: *t* is the time between the surveys; 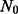 and 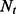 are, respectively, the initial number of individuals and the final number of individuals; 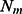 and 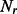 are, respectively, the number of dead trees and recruits.

To determine if the patch extinction in the PEQRB (fire protected area) was related to the size of the patch (size of host), we distributed the hosts in five height classes: 2–4 m; 4.1–6 m; 6.1–8 m; 8.1–10 m; 10.1–12 m. We used the height as surrogate for crown size, since the tallest individuals showed the largest crown areas. After that, we used ANOVA to determine if the patch extinction (death of the tree) was independent on patch size class (null hypothesis). To determine effects of mistletoe load on patch extinction, we applied a Binomial Generalized Linear Model. In this model, we used “0” for dead patches during surveys, and “1” for lived patches at the end of surveys. We also used ANOVA with a posteriori Tukey test to evaluate if the mortality of *P. robustus* per host height class in the area CZ after the fire was also independent on the host size. Since we did not find mistletoes in the height class 10.1–12 m in this area, we excluded this height class from statistical model. The analyses were performed using R statistical software [38] and the Vegan package [39].

### Ethics Statement

No specific permissions were required for this study. All owners of the areas where the data was collected were contacted and allowed the activities concerned with the project. No field work caused any further threats to endangered species.

### Sharing Materials and Data

Data deposited in the Dryad Repository: http://dx.doi.org/10.5061/dryad.jk12v.

## Results

### Dynamic Rates

We recorded, in the first survey, 752 individuals of *V. thyrsoidea* and 499 of *P. robustus* in the three studied areas (Table 1). In the last survey the number of individuals was 744 of *V. thyrsoidea* and 405 of *P. robustus* (Table 1). From the first to the last surveys, there was a decrease in the number of individuals of both species in all the areas but CE, where the number of *V. thyrsoidea* increased (Table 1).

**Table 1:** Abundance of individuals of *Psittacanthus robustus* (Loranthaceae) and *Vochysia thyrsoidea* (Vochysiaceae) in the 1^st^ and last surveys in the study areas.

Areas	*Vochysia thyrsoidea*		*Psittacanthus robustus*	
	Number of individuals in the 1^st^ survey	Number of individuals in the last survey	Number of individuals in the 1^st^ survey	Number of individuals in the last survey
PEQRB	267	257	196	177
CE	303	309	52	43
CZ	182	178	251	185

PEQRB (Parque Ecológico Quedas do Rio Bonito – six surveys; area without fire), CE (Carrancas Esmeralda – three surveys; fire before the study) and CZ (Carrancas Zilda – three surveys; fire during the study).

The rate of equilibrium of patches occupied (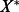) (Table 2) indicated that the local population extinction was larger than the colonization of new patches for the three sites and in most of surveys. The exception were the area PEQRB in the 4^th^ survey (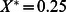) and CE in 3^rd^ survey (X* = 1) that had larger colonization than extinction (Table 2).

**Table 2:** Metapopulation parameters calculated for the mistletoe *Psittacanthus robustus* (Loranthaceae) for the study areas.

Areas	*e_h_*	*e_d_*	X*
PEQRB: 2^nd^ survey	0	9	−3.50
PEQRB: 3^rd^ survey	1	9	−0.67
PEQRB: 4^th^ survey	1	5	0.25
PEQRB: 5^th^ survey	3	4	−0.40
PEQRB: 6^th^ survey	3	3	−2.00
CE: 2^nd^ survey	5	0	0
CE: 3^rd^ survey	0	0	1.00
CZ: 2^nd^ survey	11	3	−13.00
CZ: 3^rd^ survey	1	1	0

*eh* is the number of loss of local populations on patches due to demographic or exogenous causes; *e_d_* is the loss of local population due to patch extinction and X* is the equilibrium proportion occupied. PEQRB: Parque Ecológico Quedas do Rio Bonito, area without fire; CE: Carrancas-Esmeralda, fire before the study; CZ: Carrancas-Zilda, fire during the study.

The causes for the extinction of mistletoe local populations differed among the sites. In the area without fire – PEQRB –78.9% of the local population extinction was due to patch extinction (*ed*). In the areas CE and CZ, 100% and 75%, respectively, of the extinction of mistletoe local populations occurred due to the exogenous factor fire (Table 2).

The net change rate was higher for the mistletoe population than for the host population (habitat patches) (Table 3). The net change rate values for the host in the area PEQRB varied from negative to positive and null values along different intervals, resulting in a negative balance in terms of number of individuals. CZ also showed a negative balance for the host individuals, resulting from a negative and null net change rate in consecutive intervals. CE had a positive balance in terms of host numbers, resulting of consecutive positive net change rates along the intervals. The highest net change rates for the mistletoe were found for the area CZ (Table 3). This area was burned between the 1^st^ and 2^nd^ survey, leading to the death of many mistletoe individuals, which resulted in high mortality rate and a negative net change rate (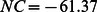 ind.yr^−1^). After that, between the 2^nd^ and 3^rd^ survey, the rate of colonization (recruitment) was much higher than mortality, resulting in a fast increase of the mistletoe population (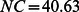 ind.yr^−1^). The net change rate in terms of number of mistletoe individuals in PEQRB fluctuated along the intervals, being sometimes negative and others positive. In CE, after a strongly negative net change rate in the first interval, the number of mistletoe did not change in the second period.

**Table 3:** Rates of mortality (M), recruitment (R) and net change (NC) for the studied species.

Areas	Mortality (M) (ind.yr^−1^)	Recruitment (R) (ind.yr^−1^)	Net change (NC) (ind.yr^−1^)
PEQRB: 1^st^ interval (*V. thyrsoidea*)	6.63	0	−6.63
PEQRB: 2^nd^ interval (*V. thyrsoidea*)	6.85	10.36	3.91
PEQRB: 3^rd^ interval (*V. thyrsoidea*)	3.77	0.77	−3.02
PEQRB: 4^th^ interval (*V. thyrsoidea*)	3.06	3.06	0
PEQRB: 5^th^ interval (*V. thyrsoidea*)	2.30	0.77	−1.54
PEQRB: 1^st^ interval (*P. robustus*)	32.13	19.30	−15.89
PEQRB: 2^nd^ interval (*P. robustus*)	39.21	46.68	14.02
PEQRB: 3^rd^ interval (*P. robustus*)	32.74	38.72	9.75
PEQRB: 4^th^ interval (*P. robustus*)	27.15	15.64	−13.64
PEQRB: 5^th^ interval (*P. robustus*)	26.26	21.21	−6.41
CZ: 1^st^ interval (*V. thyrsoidea*)	4.347	0	−4.34
CZ: 2^nd^ interval (*V. thyrsoidea*)	2.23	2.23	0
CZ: 1^st^ interval (*P. robustus*)	72.76	29.48	−61.37
CZ: 2^nd^ interval (*P. robustus*)	45.66	36.86	40.63
CE: 1^st^ interval (*V. thyrsoidea*)	0.66	2.59	1.99
CE: 2^nd^ interval (*V. thyrsoidea*)	0	1.93	1.97
CE: 1^st^ interval (*P. robustus*)	34.76	4.59	−31.62
CE: 2^nd^ interval (*P. robustus*)	9.08	9.08	0

*Psittacanthus robustus* and *Vochysia thyrsoidea* for each survey in the three studied areas. PEQRB: Parque Ecológico Quedas do Rio Bonito, area without fire; CE: Carrancas-Esmeralda, fire before the study; CZ: Carrancas-Zilda, fire during the study.

### Patch Extinction

**Figure 1:**
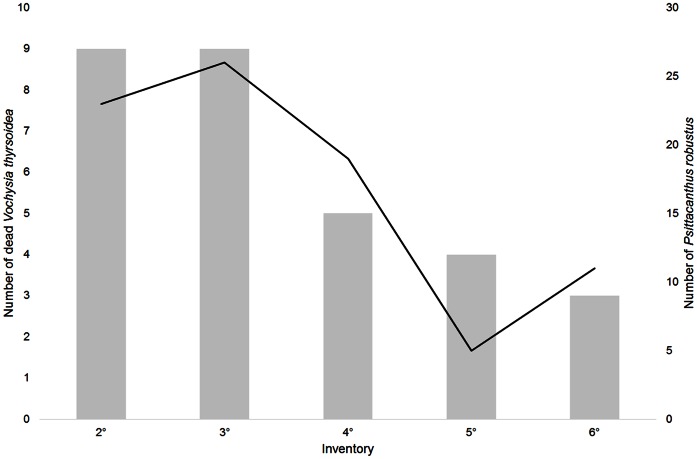
Number of mistletoe per host dead. Number of dead individuals of *Vochysia thyrsoidea* (patch extinction) in each survey in the area Parque Ecológico Quedas do Rio Bonito (PEQRB) – area without fire. The circle represents the total number of mistletoe (*Psittacanthus robustus*) in the dead individuals of *V. thyrsoidea* in each survey.

**Figure 2:**
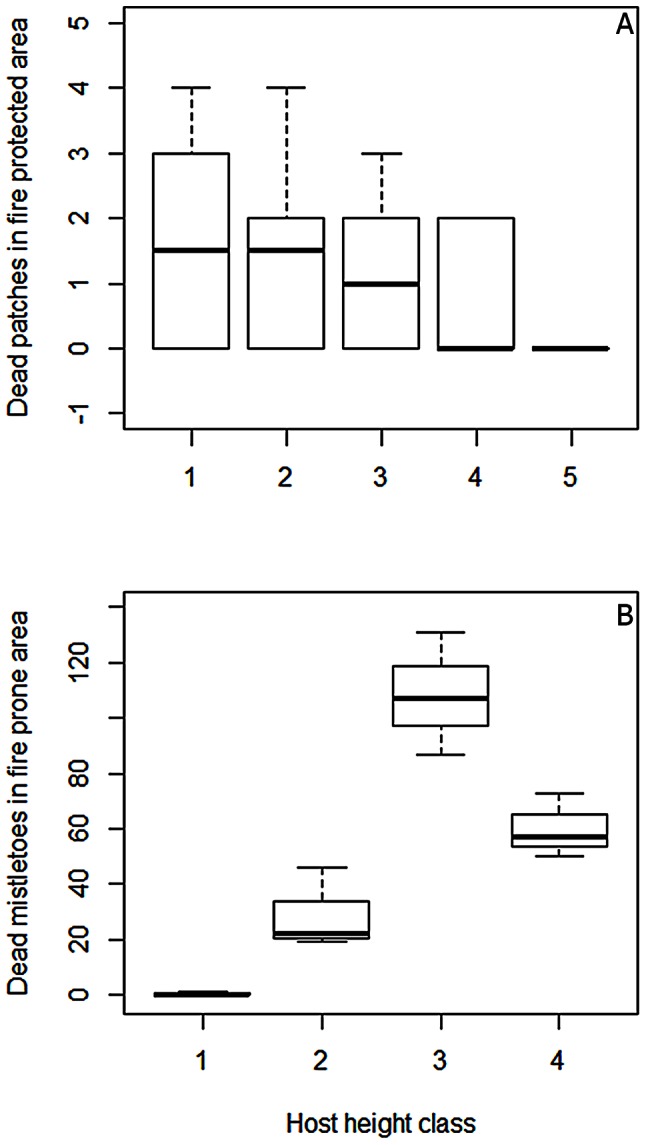
Patch extinction and mistletoe mortality per height class. **A**. Patch extinction in fire protected area Parque Ecológico Quedas do Rio Bonito (PEQRB) per class of host height (ANOVA, F_1,28_ = 7,761; p<0.009). 1: (2–4m); 2: (4.1–6m); 3: (6.1–8m); 4: (8.1–10m); 5: (10.1–12m). **B**. Mortality of *Psittacanthus robustus* in fire prone area Carrancas Zilda (CZ) per host height class (ANOVA, F_1,10 = _9.18; p = 0.01). The fire occurred between the 1^st^ and 2^nd^ surveys. 1: (2–4m); 2: (4.1–6m); 3: (6.1–8m); 4: (8.1–10m).

In the CE area, only one *V. thyrsoidea* individual died during the study period. This plant had no mistletoe. In the CZ area, six *V. thyrsoidea* died during the study. These patches housed eight *P. robustus* individuals. We found the largest mortality of *V. thyrsoidea* in the area without fire (PEQRB) where all the dead individuals held mistletoe individuals (Figure 1). Most of the patches that became extinct contained more than one mistletoe individual and all the 30 extinct patches contained a total of 84 parasite individuals. The number of patch extinctions was different between height classes (ANOVA, 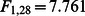; 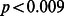; Figure 2A), however there was no effect of mistletoe load on patches extinctions (Binomial GLM, 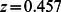; 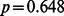).

### Fire Effect

The CE and CZ areas were burned during or just before the study. The CE area was burned before the first survey, which possibly explains the small number of mistletoes found (Table 1). In this area, there were indications that the mistletoe population was larger previously, such as marks of appressoria on *V. thyrsoidea* individuals, and dead mistletoes. All the mistletoes that remained alive in this area were attached to taller individuals on the crown apex and infested individuals were near rocky outcrops, which may have represented an additional protection against fire, possibly due to the smaller amount of combustible material.

In the CZ area the fire took place between the 1^st^ and 2^nd^ surveys, leading to a serious death toll on the mistletoes in the different height class we analyzed (ANOVA, 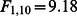; 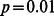; Figure 2B). In this ANOVA model, the significant differences occurred between the 1^st^–3^rd^ (Tukey, 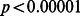), 1^st^–4^th^ (Tukey, 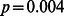), 2^nd^–3^rd^ (Tukey, 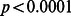), and 3^rd^–4^th^ height class (Tukey, 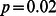). The third height class (6.1–8 m) exhibited the highest mortality of *P. robustus* (Figure 2B).

## Discussion

The structure and dynamics of *P. robustus* population on *V. thyrsoidea* population fitted the metapopulation premises proposed by Hanski [14]: (i) the suitable habitats for the mistletoe (host trees) occur in discrete patches which were occupied by local populations; (ii) local populations went extinct during the study and (iii) colonization of previously non-occupied patches occurred. The fourth premise states that the local populations did not exhibit synchronous dynamics, with different colonization and extinction over time. Possibly the mistletoe populations fail to fulfill this premise because the fire events tend to cause synchronous mortality and posterior recruitment in local populations in a specific area. On the other hand, this synchronism is not an intrinsic population trait, but instead the result of an exogenous factor.

*Psittacanthus robustus* fitted the incidence function model [14], [15], [16] as the most important events governing the population dynamics were related to colonization and extinction rates in the patches. These events depend on patch size and the population size in each patch. The patch size is an important variable for the hemiparasites. The largest individuals of *V. thyrsoidea* supported the largest *P. robustus* local populations [34]. This pattern is common for hemiparasites that have been dispersed by birds [40]. According to Overton [11] the relationship between host size and infection intensity occurs because larger trees are older and have more time and higher probability of being colonized. In addition, the behavior of the disperser bird is a key factor in the deposition of mistletoe seeds [26]. These birds prefer to forage in taller individuals with dense canopies [5]. Finally, there is the area effect, where larger individuals (patches) offer more branches (space) for mistletoe colonization.

### Patch Extinction and Fire Effect

In the three studied areas, the number of occupied patches declined with time. In the PEQRB area, this fact was related with patch extinction caused by the parasitism. As we expected, the mistletoe abundance was associated to the patch habitat dynamics, where mistletoe infestation seems to lead to the death of the host. In the area PEQRB, where fire was absent, all the 30 patches that went extinct were occupied by mistletoe. The parasitism interferes with in host species fitness, altering the competitive interaction between the host plants and non-host plants [41]. The cumulative impacts of a long and large infestation can result in severe pathogenic effects to the host [4] and, in extreme cases; the high mistletoe intensity can result in host death [27].

In the CE and CZ areas, the fire caused a high local extinction of *P. robustus* or reduced the mistletoe parasitism per habitat patch and, probably due to this effect, the mortality of *V. thyrsoidea* was almost zero in those areas. The species *P. robustus* is distributed in Cerrado areas [5], where the fire is a natural and recurrent event. However, this species is fire sensitive. On the other hand, *V. thyrsoidea* is fire tolerant. Periodic burning seems to be an important factor for controlling infestation, which can increase the probability of survival of the host.

In a study with *Pinus* sp. in a temperate forest, the natural fire controlled the infection rate by the mistletoe *Arceuthobium americanum* [42]. The authors found that the infection intensity was not a direct result of fire suppression, even though long term fire protection could lead to the increase of abundance of the hemiparasite. In an experimental study evaluating the fire effect and thinning on the intensity of *Arceuthobium* spp. infection on the host species *Pinus ponderosa* and *Pseudotsuga menziesii*, the fire reduced the infestation severity in all treatments [43]. In Australia and North America, the increase in mistletoe populations was attributed to the reduction of fire frequence [9], [44], [45] because of many hemiparasite species were fire intolerant.

### Metapopulation Dynamics of *Psittacanthus robustus* and their Implications

Snäll et al. [18] proposed a generalist model of spatial-temporal dynamics: habitat-tracking metapopulation model. The model was originally based on studies with epiphytic bryophytes, but can be applied to other groups [30], [46], [47]. In this system, the characteristics of host trees, as well as the environmental conditions and connectivity, were important for explaining the distribution patterns of bryophytes in forest environments [21], [22]. In the tree-epiphyte system, first the trees are established in the environment, and then the colonization by epiphytes begins. Therefore, the oldest trees should have a larger epiphyte population due a longer exposition to the colonization process. These patches would become sources of propagules to other patches. In the habitat-tracking metapopulation model, patch extinction occurs regardless of the occurrence or abundance of epiphytes. In their model, the epiphytes do not interfere on the patch survival [18], [21].

Mistletoes seem to follow a process similar to epiphytic bryophytes, however with some differences. Although the mistletoes have an aggregated distribution in habitat patches similar to the pattern observed for epiphytes [5], [18], [34], the *Psittacanthus robustus* population does seem to affect the patch survivorship, since infested individuals of *V. thyrsoidea* had a higher mortality than those without mistletoes. The *P. robustus* population size increased with the size of the host tree (patch) [32], which is a premise in the metapopulation model [15], [16], [18]. However, in the mistletoe case, an increase in population size also increases the risk of local extinction by the patch eradication. Therefore, we propose the inclusion of the effect of local population on the patch existence in a patch-tracking metapopulation model applied to parasitic epiphytes.

Although the studied system does fit to the expected general premises for metapopulations, it also points to other aspects that may be important for some species with metapopulation structure. For the mistletoes studied here, the size of the tree is very important, since larger trees have larger local populations and larger and taller trees offer a better protection against fire [34]. The effect of the increase of population size on the decrease of its chance of extinction is a well-established principle on population ecology [48], [49]. On the other hand, our results point out that the size of the patch (host tree) can have an effect that goes beyond the simpler assumption of a large local population. Larger trees also buffer the local mistletoe population against destructive fire events. That kind of effect must be also present in other situations, since larger patches have a lower relationship perimeter/area and that buffers the local population against external influences [50].

The negative effects of mistletoe infestation on the patch existence (host survivorship) should also be considered in a broader perspective. One another possible example of this kind of effect is the metapopulational dynamic of species populations specialized on occupation of recently formed gaps in forests. A recent gap in a forest provides a strong increase of light conditions on the ground, a resource otherwise scarce in the forest understory. Those discreet gaps offered unique opportunities for light demanding species establish themselves and, therefore, those species show a metapopulation structure and dynamics [51]. On the other hand, the same species that colonize gaps, alone or combined with other light demanding species, are also responsible for reducing light resources posteriorly and turning the patches unsuitable to their own offspring [51]. Therefore, we suggest that those effects of local population on patches existence or states must be incorporated in a patch-tracking metapopulation model [18].

Finally, frequently metapopulations are analyzed in a framework of equilibrium state between extinction and recolonization of patches [11], [17]. For the studied mistletoe, this is probably an unrealistic assumption, since fire events tend to generate periods of intense extinction followed by elevated recolonization originated from the surviving patches (larger local populations and higher trees). This patch dynamic seems similar to source-sink model considered by Freckleton and Watkinson [17] as a “true” variation of metapopulation behavior, where the larger and probably older trees work as source and the smaller and probably younger ones work as sink.
